# Metagenomic Analysis of Cucumber RNA from East Timor Reveals an *Aphid lethal paralysis virus* Genome

**DOI:** 10.1128/genomeA.01445-16

**Published:** 2017-01-12

**Authors:** Solomon Maina, Owain R. Edwards, Luis de Almeida, Abel Ximenes, Roger A. C. Jones

**Affiliations:** a School of Agriculture and Environment, Faculty of Science, University of Western Australia, Crawley, Western Australia, Australia; b Institute of Agriculture, Faculty of Science, University of Western Australia, Crawley, Western Australia, Australia; c Cooperative Research Centre for Plant Biosecurity, Canberra, Australian Capital Territory, Australia; d CSIRO Land and Water, Floreat Park, Western Australia, Australia; e Seeds of Life Project, Ministry of Agriculture and Fisheries, Dili, East Timor; f DNQB-Plant Quarantine International Airport Nicolau Lobato Comoro, Dili, East Timor; g Department of Agriculture and Food Western Australia, South Perth, Western Australia, Australia

## Abstract

We present here the first complete genomic *Aphid lethal paralysis virus* (ALPV) sequence isolated from cucumber plant RNA from East Timor. We compare it with two complete ALPV genome sequences from China, and one each from Israel, South Africa, and the United States. It most closely resembled the Chinese isolate LGH genome.

## GENOME ANNOUNCEMENT

As part of a project to examine possible connectivity between viruses infecting crops in northern Australia and nearby Southeast Asian countries, virus genomes from plant samples from East Timor and Australia were compared (1–6). During July and September 2015, 15- and 21-cucurbit leaf samples were collected in East Timor and Broome in northwest Australia, respectively, and subjected to next-generation sequencing. A complete genome of *Aphid lethal paralysis virus* (ALPV) was obtained from East Timorese cucumber (*Cucumis sativus*) sample TM19, but not from any other samples. ALPV belongs to the genus *Cripavirus*, family *Dicistroviridae*. It was first identified in 1988 in South Africa and contains a 9.7-kb polyadenylated ssRNA (7, 8). Five complete ALPV genomes are available in GenBank: two from China, and one each from the United States, Israel, and South Africa (9, 10). Metagenomics offers prospects for discovering novel viruses with distinct genome ontologies (11–16). RNA-Seq with rRNA-Plant depletion provides reliable metagenomic detection of polyadenylated and nonpolyadenylated RNA viruses (17). This approach detected ALPV in sample TM19.

The 15 East Timorese samples were blotted onto Fast Technology for Analysis of nucleic acids (FTA) cards (18) before dispatch to Australia. The Australian samples studied were recently collected leaves. Total RNA was extracted from both sample types using a ZR plant RNA mini prep kit (Zymo Research). The total RNA extracts were treated with RNase-free DNase (Invitrogen) and measured using Qubit (Invitrogen). RNA integrity was confirmed using RNA screen Tape (TapeStation 2200, Agilent Technologies). Libraries were prepared from total RNA using a TruSeq stranded Total RNA sample preparation Ribo-Zero Plant kit (catalogue no. RS-122-2401, Illumina) (1–6). Sequencing was by MiSeq using a V2 kit (Illumina) with 2 × 151 cycles of paired-end reads. Reads were assembled and genomes annotated using CLC Genomics Workbench version 6.5 (CLC bio) and Geneious version 8.1.7 (Biomatters) (19). Further alignment was by MAFFT (20).

FTA card sample TM19 yielded 2,287,025 reads and, after trimming, 2,248,678 remained. *De novo* assembly generated 270 contigs and 858,904 reads mapped to the contig of interest with coverage of 30,410×. The final complete genome length was 9,789 nucleotides (nt). As with other members of the genus *Cripavirus*, it coded for a nonstructural polyprotein (encoding putative helicase, protease, and RNA-dependent RNA polymerase) and a capsid protein precursor (encoding proteins VP1 to VP4). A BLAST-based search with the pairwise sequence comparison (PASC) tool (21), revealed that the TM19 genome sequence most resembled Chinese isolate LGH (accession no. KR021407) with 93.8% nt identity. As mentioned above, the purpose of these studies was to search for plant rather than insect viruses. Possibly, ALPV resembles a related aphid virus, *Rhopalosiphum padi virus*, in spreading horizontally from the initial viruliferous aphid feeding site rapidly via the phloem, allowing nonviruliferous aphids to acquire it throughout the plant (22), which might explain its detection in a cucumber sample.

### Accession number(s).

This sequence was deposited in GenBank under the accession number KX830963.
